# Subtle reproductive impairment through nitric oxide-mediated mechanisms in sea urchins from an area affected by harmful algal blooms

**DOI:** 10.1038/srep26086

**Published:** 2016-05-19

**Authors:** Oriana Migliaccio, Immacolata Castellano, Davide Di Cioccio, Gabriella Tedeschi, Armando Negri, Paola Cirino, Giovanna Romano, Adriana Zingone, Anna Palumbo

**Affiliations:** 1 Stazione Zoologica Anton Dohrn, Naples, Italy; 2 D.I.P.A.V. – Section of Biochemistry, University of Milan, Milan, Italy

## Abstract

The health of the sea urchin *Paracentrotus lividus*, a key species in the Mediterranean Sea, is menaced by several pressures in coastal environments. Here, we aimed at assessing the reproductive ability of apparently healthy *P. lividus* population in a marine protected area affected by toxic blooms of *Ostreospsis* cf. *ovata*. Wide-ranging analyses were performed in animals collected prior to and during the bloom, as well as at several times thereafter, during the reproductive season. Adults showed a low fertilization rate, along with high nitric oxide (NO) levels in the gonads and the nitration of the major yolk protein toposome, which is an important player in sea urchin development. Serious developmental anomalies were observed in the progeny, which persist several months after the bloom. NO levels were high in the different developmental stages, which also showed variations in the transcription of several genes that were found to be directly or indirectly modulated by NO. These results highlight subtle but important reproductive flaws transmitted from the female gonads to the offspring with the NO involvement. Despite a recovery along time after the bloom, insidious damages can be envisaged in the local sea urchin population, with possible reverberation on the whole benthic system.

In the Mediterranean Sea, the sea urchin *Paracentrotus lividus* represents a key species in the benthic communities, where it controls the dynamic, structure and composition of shallow macroalgal assemblages through its grazing activity^1^. In addition, *P. lividus* represents a food source for fishes and other animals, including humans that consider its gonads a culinary delicacy. The health of *P. lividus* and its safety as food are therefore of great interest at multiple levels. Nevertheless sea urchins continuously face the impact of a variety of increasing pressures, acting single or simultaneously in the coastal environments, including eutrophication, ocean acidification, warming, hypoxia, chemical contamination and harmful algal blooms (HABs). These impacts are more deleterious for benthic organisms because of their limited or null motility, and in case of abundant or structuring species may have detrimental consequences on the biodiversity and functionality of the whole benthic system^2^.

Among HABs are blooms of species of the benthic dinoflagellate genus *Ostreopsis*, which produces ovatoxins^3^,^4^,^5^, i.e. palytoxin- like molecules that are deemed to be responsible for a number of health problems in humans^6^ (for a review). *Ostreopsis* cf. *ovata*, first reported in subtropical waters^7^, has been increasingly recorded also in temperate seas and in the Mediterranean Sea^8^, with intense blooms in July/August in the western and in September-October in the Adriatic Sea, (e.g.^9^) and in the eastern basin^10^. Occasionally, mortality of benthic organisms, including sea urchin, or damages to the exoskeleton have been reported during *O.* cf. *ovata* blooms^11^,^12^, whereas fertilization impairment and embryo/larvae mortality were observed upon exposure to *Ostreopsis* cultures^13^,^14^.

In the Mediterranean Sea, the reproductive cycle of *P. lividus* generally starts in autumn and has a main peak in spring^15^, which limits the possibility of a direct impact of *Ostreopsis* blooms on the reproduction. However, *Ostreopsis* bloom might have long-term effects on the reproductive ability of natural sea urchin population inhabiting highly affected sites. This aspect is relevant considering the potential decline of this important species as a consequence of environmental changes, predation and overexploitation^16^,^17^. In the Gulf of Naples (Tyrrhenian Sea, western Mediterranean Sea), *O*. cf. *ovata* was first detected in 2005^18^, and hence regularly monitored since 2007. Within the area, the highest frequency and intensity of the blooms are recorded in the Gaiola Marine Protected Area (MPA), a hotspot of marine biodiversity. Ovatoxins have been detected in many cases in sea urchins or mussels^19^, but mortality of benthic animals has never been reported in relation with *O*. cf. *ovata* blooms in the Gulf of Naples.

In this study, we aimed at elucidating the reproductive functionality of a *P. lividus* population inhabiting the Gaiola MPA. Sea urchins were sampled previous to the bloom, at the peak of the bloom and some months thereafter, on three dates along the reproductive season. Spawning, fertilization and development were followed in the laboratory. Our wide-ranging approach included biochemical analysis of the gonads, reproductive success, morphological and biochemical analysis of the sea urchin progeny along with selected gene expression profile. In addition, the role of nitric oxide (NO) in *P. lividus* reproduction and offspring development was explored, considering the pivotal biological functions of NO in regulating fundamental processes in marine organisms^20^,^21^,^22^ and in mediating the response of *P. lividus* to several stress agents, including metal ions and toxic diatom aldehydes^23^,^24^,^25^.

## Results

### *Ostreopsis* and sea urchins from the MPA Gaiola site

*Ostreopsis* and sea urchins were collected at the MPA Gaiola previous to the bloom (June 2012), during the bloom (July 2012, 2013), and at several times thereafter, during the reproductive season (October 2013, February and April 2014), thus capturing blooms of the toxic microalga in 2012 and 2013.

As controls, sea urchins were collected at the different times at Castel dell’Ovo, another site of the Naples coast (Supplementary Fig. S1) which is known to harbour *O.* cf. *ovata* in very low concentrations during summer.

At MPA Gaiola, in the pre-bloom phase (June 2012), *O.* cf. *ovata* densities were lower than 115 cells g^−1^ macroalgal fresh weight (fw). The blooms (July 2012 and 2013) showed peaks of up to 1.6 × 10^5^ cells g^−1^macroalgal fw. Since August, concentrations dropped to less than 10^4^ cells g^−1^ macroalgal fw, whereas values lower than of 5 × 10^3^ cells g^−1^macroalgal fw were recorded in autumn. *Ostreopsis* cf. *ovata* sampling was suspended later in the year until next spring because only rarely a few cells have been recorded in this period.

Chemical analyses (MS-TOF) of the extracts from sea urchins collected at Gaiola revealed a concentration of 78.8 and 80 μg/kg ovatoxin-a in July 2012 and 2013, respectively, whereas the toxin was not found in the sea urchins collected in other periods of the year. No palytoxin-like toxins were detected in sea urchins collected at the control site Castel dell’Ovo during the study period.

Sea urchins collected at MPA Gaiola were visually examined for movement ability, spine losses and feeding behavior. They looked generally healthy, only occasionally showing loss of spines, with no apparent differences from animals from Castel dell’Ovo.

### Reproductive ability of *P. lividus* at the MPA Gaiola site

**Figure 1:**
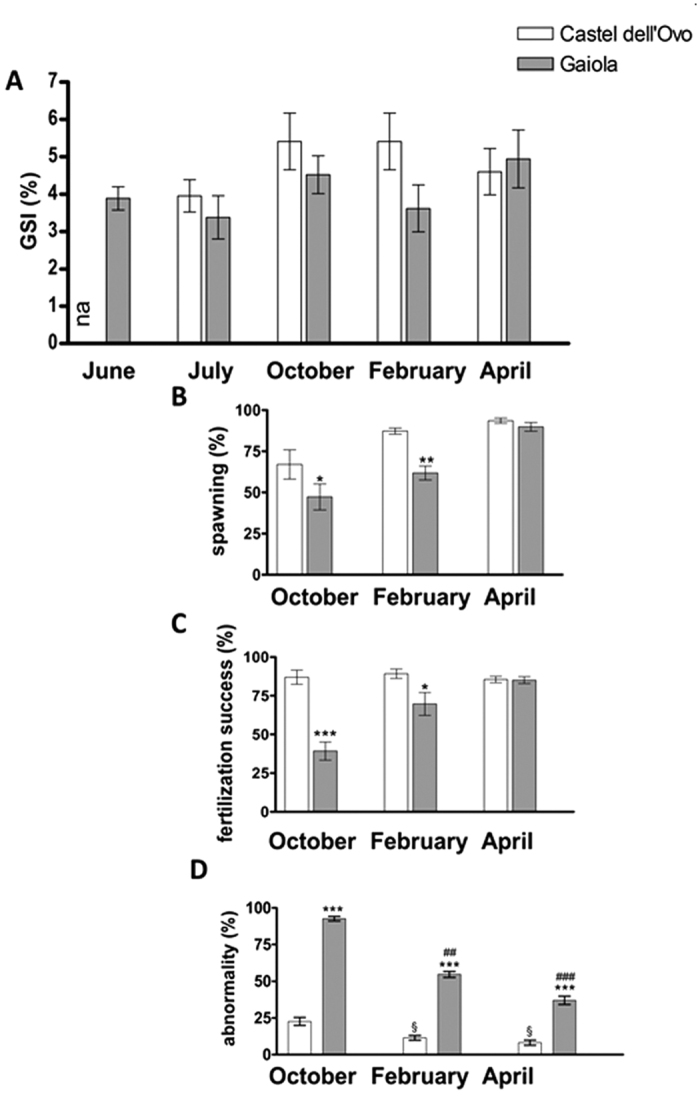
Sea urchin reproductive state and offspring morphology at the Gaiola MPA site. Female sea urchins collected at Gaiola site previous to the *O.* cf. *ovata* bloom (June), at the peak of the bloom (July) and after the bloom (October, February and April) were examined for GSI (**A**). Female sea urchins collected at Gaiola site in the reproductive period (October, February and April) were examined for spawning (**B**), fertilization success (**C**) and abnormal plutei (**D**), as described in Methods. Control animals were collected from the site of Castel dell’Ovo. Data are reported as mean ± SEM. na = non available data. *Significant difference from Castel dell’Ovo specimens: *P < 0.05, **P < 0.01, ***P < 0.001. ^#^Significant difference compared to October (Gaiola): ^##^*P* < 0.01; ^###^*P* < 0.001. ^§^Significant difference compared to October (Castel dell’Ovo): ^§^*P* < 0.05. Two-way ANOVA, with Bonferroni’s post hoc test for normally distributed data (**A**,**C**,**D**) and Kruskal-Wallis test (K/S) with Mann-Whitney pairwise post hoc test for not normally distributed (**B**) (*P* < 0.05). N = 15 (**A**–**C**) and N = 6 (**D**).

The gonadosomatic index (GSI) values did not differ significantly between sea urchins collected at Gaiola MPA and Castel dell’Ovo (Fig. 1A). However, in October, spawning percentage and fertilization success of sea urchin females from Gaiola MPA were considerably lower than those of the control site (Fig. 1B,C). In February, these parameters were still low, whereas they were similar to the control values in April (Fig. 1B,C).

A high number of embryos from animals collected at Gaiola showed abnormal features, which consisted of defects in arm and skeleton elongation, reduced embryo dimension and developmental delay (Fig. 1D). In October, anomalies affected up to 92% of the embryos and were accompanied by important developmental delay. Most of the embryos analyzed were indeed at the blastula or gastrula stage, compared to the pluteus stage of the control, which consisted of cone-shaped larvae with four fully developed arms and complete skeletal rods. In February offspring, anomalous embryos were less (55.66 ± 5.08%) while impairments were mainly found in arms and apex. The percentage of abnormal embryos further decreased in April offspring (37.00 ± 6.78%), when anomalies only consisted of smaller embryos often with abnormal arms. A reduction over time in the percentage of abnormal embryos (from 25 to 8%) was also observed in the offspring of control animals from Castel dell’Ovo, as normally observed along the reproductive season of *P. lividus*.

### Gene expression profile of *P. lividus* offspring from the MPA Gaiola site

To further characterize the progeny of *P. lividus* from the MPA Gaiola, the expression level of selected genes was analyzed by Real Time qPCR in different developmental stages of the offspring obtained in the laboratory from females collected at Gaiola MPA in October, February and April. The examined genes are involved in different functional responses, including stress (*hsp70, hsp60, hsp56*), skeletogenesis (*sm30, sm50, bmp5-7, msp130, p16, p19, fg9/16/20*), multidrug efflux (*abc1b, abc4a, abc8b, abc1a*) and NO production (*nos*).

**Figure 2:**
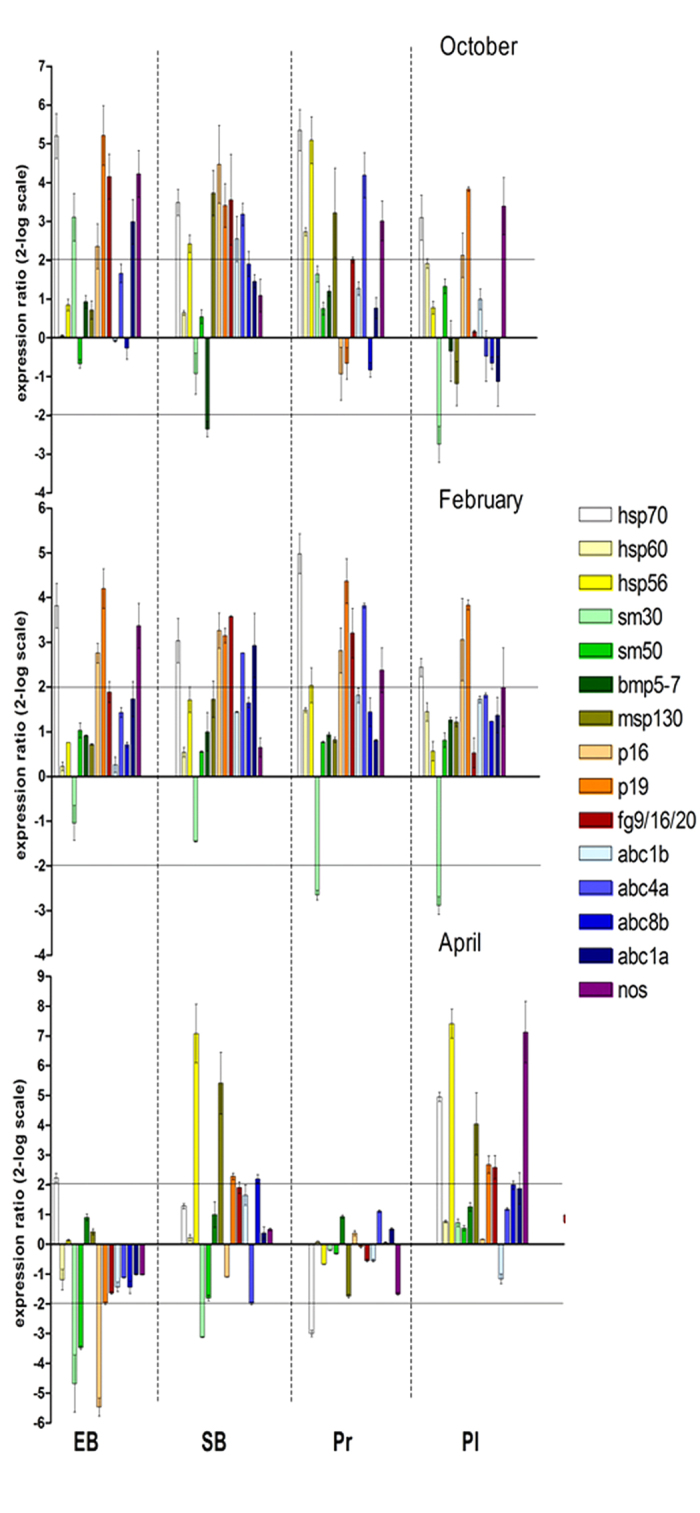
Gene expression of the progenies of *P. lividus* from the Gaiola MPA. Eggs from sea urchin females collected at Gaiola site some months after *O.* cf. *ovata* bloom, i.e. in October, February and April, were fertilized with mixed sperm from 3 males from the control site of Castel dell’Ovo. Different developmental stages (early blastula EB, swimming blastula SB, prism Pr and pluteus Pl) were examined for the expression of selected genes by Real Time qPCR. Data are reported as fold difference in the expression levels of the analyzed genes, compared to control (mean ± SEM) represented by offspring of females collected from Castel dell’Ovo. Fold differences greater than ±2 (horizontal guidelines) were considered significant.

In all cases, the gene expression profile was altered in the offspring from Gaiola animals (Fig. 2). In October, many genes were up-regulated: *hsp70* at all stages, *p16* and *p19* at the early blastula, swimming blastula and pluteus stages, *fg9/16/20* at the early blastula, swimming blastula and prism stages, *nos* at the early blastula, prism and pluteus stages, *hsp56, msp130* and *abc4a* at the swimming blastula and prism stages, *hsp60* at the prism stage, *abc1a* at the early blastula stage, *abc1b* at the swimming blastula stage. The gene *bmp5-7* was down-regulated at the swimming blastula stage, while *sm30* was up-regulated at the early blastula stage and down-regulated at the pluteus stage. In February, the offspring showed a lower number of genes affected compared to October. Yet some genes were upregulated: *hsp70, p16* and *p19* at all developmental stages, *fg9/16/20* at the swimming blastula and prism stages, *nos* at the early blastula, prism and pluteus stages, *abc4a* at the swimming blastula and prism stages, *abc1a* at the swimming blastula stage. The gene *sm30* was down-regulated at the prism and pluteus stages. In April, with a lower percentage of abnormal plutei, the number of genes altered was still lower, but their expression profile often differed from that of the October and/or February progeny. For example in some stages *hsp56, msp130* and *nos* showed a pronounced up-regulation, while *sm50* and *p16* were down-regulated at the early blastula stage.

### Toposome nitration in the female gonads of sea urchins from the MPA Gaiola site

**Figure 3:**
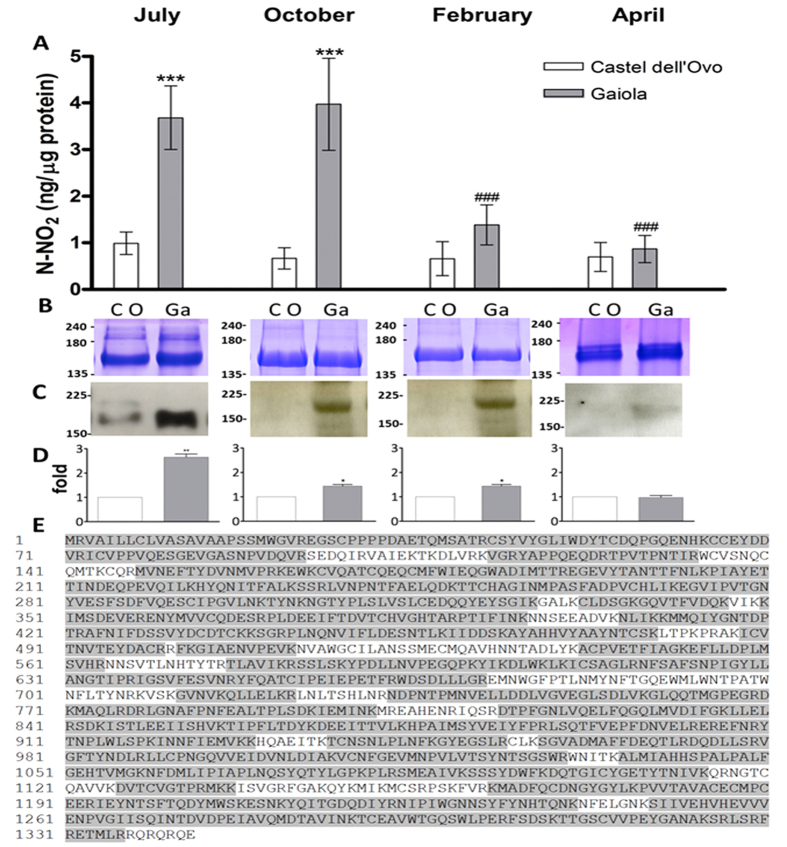
NO levels and formation of nitrated toposome in gonads of *P. lividus* from the Gaiola MPA. Ovaries of sea urchins collected at Gaiola site at the peak of the bloom (July) and after the bloom (October, February and April) were examined for NO concentration (**A**) and formation of nitrated proteins (**B**–**D**), as described in Methods. As controls, sea urchins were collected at Castel dell’Ovo (July, October, February and April). (**A**) NO levels determined as nitrite by the Griess assay. Data are reported as mean ± SEM. *Significant difference compared to the respective controls from Castel dell’Ovo: ***P < 0.001. ^#^Significant difference compared to July: ^###^*P* < 0.001. Kruskal-Wallis test (K/S) with Mann-Whitney pairwise post hoc test (*P* < 0.05). N = 8. (**B**,**C**) A representative experiment showing protein extracts examined by SDS-PAGE with Coomassie staining (**B**) and western blot with anti-nitrotyrosine antibodies (**C**). Coomassie staining was used as loading control (CO, Castel dell’Ovo, Ga, Gaiola). (**D**) Histogram showing densitometric analysis of the immunopositive band with respect to the corresponding controls from Castel dell’Ovo in 3 biological replicates, expressed as fold average increased value ± SEM. Significant difference compared to the control, *P < 0.05, **P < 0.01. One-way ANOVA, Dunnet’s post hoc test (P < 0.05). The ratio between band intensity values of exposed animal and control was assumed as 1. The bands were quantified by Java Image software. N = 5. (**E**) Identification of the immunopositive band as toposome by MS/MS analysis: protein sequence obtained by MS/MS analysis in gonads from Gaiola MPA animals collected in the July bloom phase (highlighted in grey) mapped on toposome sequence from *P. lividus* (NCBI accession number AAQ17121)^62^.

Gonads of sea urchins were analyzed for endogenous NO levels and NO-induced formation of nitrated proteins (Fig. 3). Markedly, higher NO levels were found in ovaries of sea urchins collected from Gaiola in July and October (3.68 ± 0.68 and 3.97 ± 0.98 ng nitrite/μg protein, respectively) compared to the respective controls from Castel dell’Ovo (0.99 ± 0.24 and 0.67 ± 0.23 ng nitrite/μg protein, respectively) (Fig. 3A) and in the Gaiola pre-bloom conditions in June (1.79 ± 0.11 ng nitrite/μg protein). In February and April, NO values in gonads of Gaiola animals decreased, approaching the control values. Sea urchins ovary extracts, analyzed by western blot using an anti-nitrotyrosine antibody, showed a major band at approximately 170 kDa (Fig. 3B,C,D). The level of protein nitration was higher in samples of July, decreased in October and February animals, and became comparable to the control values of animals from Castel dell’Ovo in April (Fig. 3C,D). Nitration of this major band was not detected in the gonads of sea urchins collected at Gaiola MPA previous to the bloom (June). The size of the nitrated protein corresponded to that predicted for toposome, which represents the major yolk protein in the gonads. Nitrated protein identification was confirmed by tandem mass spectrometry (Fig. 3E), which produced a sequence accounting for an 83.47% coverage of *P. lividus* toposome.

### Involvement of NO in the offspring development of *P. lividus* from the MPA Gaiola site

**Figure 4:**
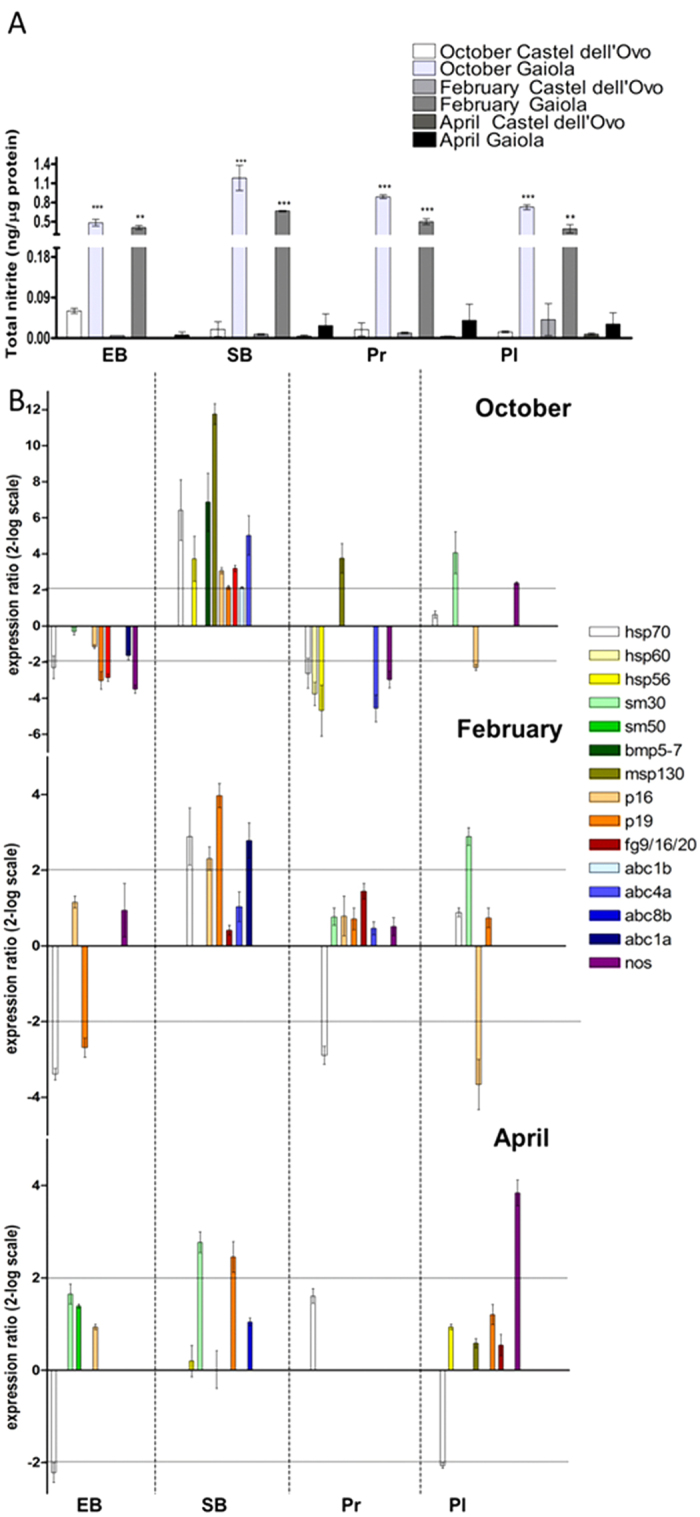
NO-modulated development of the progenies of *P. lividus* from the Gaiola MPA. Offspring of sea urchins collected at Gaiola site after *O.* cf. *ovata* bloom (October, February and April) were examined for NO concentration (**A**) and gene expression (**B**). (**A**) NO levels determined as nitrite by the Griess assay from animals collected at Gaiola site and at the control site Castel dell’Ovo. Data are reported as mean ± SEM. *Significant difference compared to the control: **P < 0.01, ***P < 0.001. Kruskal-Wallis test (K/S) with Mann-Whitney pairwise post hoc test (P < 0.05). N = 6. (**B**) The expression of the genes found to be altered at Gaiola site, as shown in Fig. 2, was followed at the different developmental stages (early blastula EB, swimming blastula SB, prism Pr and pluteus Pl) by Real Time qPCR under low NO levels in the presence of 10^−4^ M TRIM. Data are reported as fold difference compared to control (mean ± SEM), offspring of females collected from Gaiola and reared in the absence of TRIM. Fold differences greater than ±2 (see horizontal guidelines at values of 2 and −2) were considered significant.

The high NO levels in the ovaries, likely responsible for toposome nitration, prompted us to investigate the possible link between NO and the observed impairment of the fertilization and embryo development in Gaiola animals. Indeed, in the offspring of females collected from Gaiola in October and February NO levels were remarkably higher than the respective controls from Castel dell’Ovo (Fig. 4A), whereas no significant differences between the two sampling sites were found in the offspring of females collected in April.

To confirm NO involvement in developmental anomalies, the sea urchin progeny was reared in the presence of the NOS inhibitor TRIM, which reduces endogenous NO levels in developing embryos^24^. The morphology and the expression of the genes previously found to be affected (see Fig. 2) were hence analyzed in the different developmental stages. In offspring from October, February and April animals reared with TRIM, abnormal embryos increased to 100, 77 and 66%, respectively, compared to 95, 57 and 37% observed in offspring reared in normal sea water, suggesting a protective role of NO. In addition, under reduced NO conditions several genes showed modifications in expression levels, supporting NO involvement in transcriptional anomalies detected in the offspring of Gaiola animals. Despite a great variability, some genes showed the same variation trend at different sampling times (Fig. 4B). These included: *hsp70* (early blastula) and *p19* (swimming blastula) in October, February and April, *nos* (pluteus) in October and April, *p16* (swimming and pluteus) in October and February, and *sm30* (pluteus) in October and February. Other genes, i.e. *hsp56, p16, fg9/16/20* and *nos*, showed an opposite trend at different developmental stages in samples collected at the same time. Overall, the expression of *hsp70, hsp60, hsp56, sm30, bmp5*-7, *msp130, p16, p19, fg9*/*16*/20, *abc4a, abc1a, abc1b* and *nos* was affected by TRIM, suggesting a regulation of these genes by NO.

**Figure 5:**
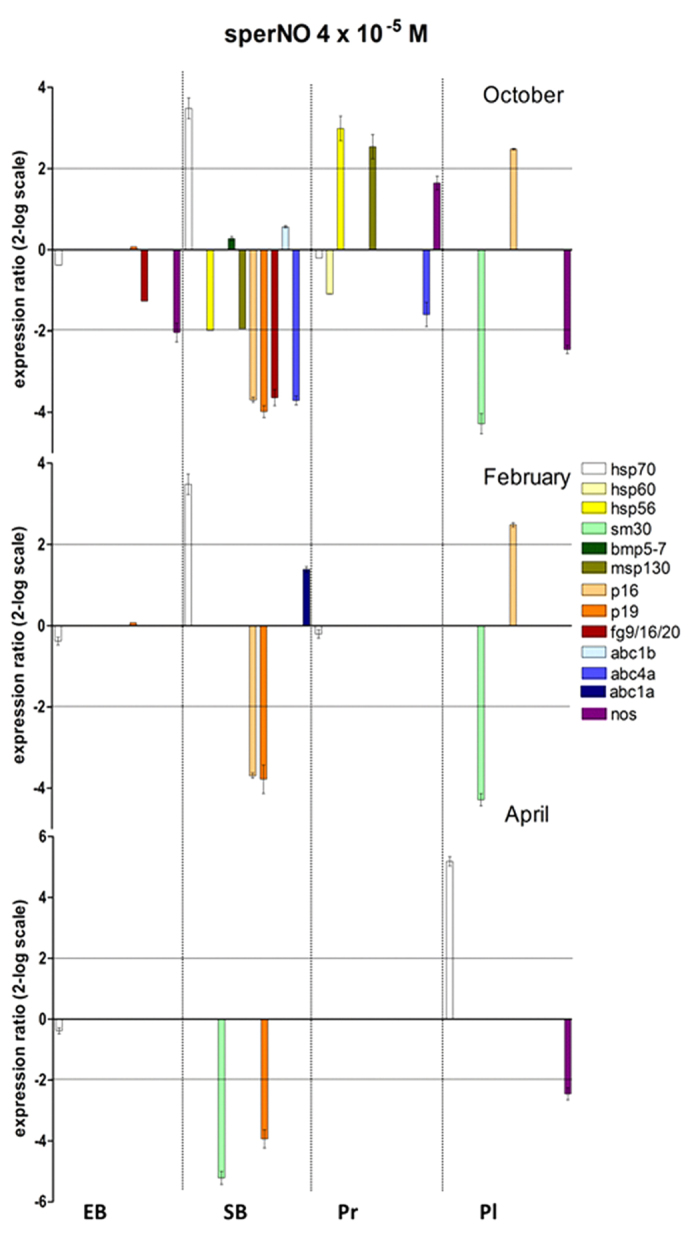
Gene expression of the progenies of *P. lividus* from the control site Castel dell’Ovo under induced high levels of NO. The expression of the genes found to be altered by TRIM treatment in Gaiola offspring, as shown in Fig. 4, was followed by Real Time qPCR in the different developmental stages (early blastula EB, swimming blastula SB, prism Pr and pluteus Pl) of the offspring from the control site reared under high NO levels in the presence of 4 × 10^−5^ M sperNO. Data are reported as a fold difference (mean ± SEM) in the expression levels of the analyzed genes compared to controls (mean ± SEM), the offspring of females collected from Castel dell’Ovo and reared in the presence of spermine, the product derived from sperNO after NO release, respectively. Fold differences greater than ±2 (see horizontal guidelines at values of 2 and −2) were considered significant.

To confirm that genes altered by TRIM were actually affected by NO, their expression as mRNA levels was measured in the offspring of control animals from Castel dell’Ovo reared in the presence of the NO donor sperNO (Fig. 5), using as a control spermine, the product derived from sperNO after NO release. The expression of *hsp70, hsp56, sm30, msp130, p16, p19, fg9*/*16*/20, *abc4a* and *nos* was considerably influenced by the NO donor in different developmental stages and at different sampling times. Notably, the expression of most genes under high NO levels (sperNO) showed an opposite trend compared to that observed under reduced NO levels (TRIM).

## Discussion

The wide ranging combination of morphological and functional observations reported in this study revealed a significant impairment of reproductive ability in natural populations of sea urchins inhabiting an MPA that is affected by intense and recurrent HABs produced by the dinoflagellate *O.* cf. *ovata*. The sampling sites of MPA Gaiola and Castel dell’Ovo were selected based on previous knowledge of the temporal and spatial distribution of *O.* cf. *ovata* in the Gulf of Naples, generally characterised by peak densities at the MPA Gaiola and very low concentrations at Castel dell’Ovo during summer. The low densities at the latter site could be due to lower abundance of the brown and red macroalgae on which the dinoflagellate grows as an epiphyte. However, the environmental variables driving the development harmful benthic microalgae blooms are not fully understood^8^. The relatively high content of toxins in animals from Gaiola MPA during the bloom in July, compared to undetectable levels in animals from Castel dell’Ovo, support the idea that sea urchins were actually exposed to the toxic dinoflagellate only at Gaiola. In addition, the correspondence between the seasonality of *Ostreospsis* bloom and sea urchin biochemical response strongly suggests an involvement of *O.* cf. *ovata* in the effects reported in this study.

Our results on sea urchin reproductive dynamics point at a subtle but important maternal-transmitted impact.Despite the immaturity of the animals at the time of the bloom, a compromised reproductive ability of *P. lividus* is evident in both the fertilization success and the progeny development process occurring several months thereafter. Not only the morphology, but also the gene expression is affected in the different developmental stages, with a partial recovery only towards the end of the reproductive period. The altered NO levels indicate that this pivotal molecule plays a key role in the transmission of the toxic effect across generations, initially being involved in processes occurring in the gonads and subsequently acting in the defense mechanisms and in the recovery of the offspring.

Trans-generational consequences of environmental contamination have been an area of recent investigation in the marine environment^26^, whereby the parental transmission of phenotypes may alter susceptibility to a stress factor and, on a long-term scale, modify population dynamics. For example, *Strongylocentrotus droebachiensis* and *Pseudocalanus acuspes*, showed a transgenerational alleviation of the effects of ocean acidification^27^,^28^. By contrast, in *Oryzias latipes, Danio rerio* and *P. lividus*, the exposure to chemicals induces transgenerational phenotypes of reproductive impairment, skeletal deformities and compromised embryo survival during the next generations^25^,^26^,^29^.

The case presented in this study, however, differs from those mentioned above because environmental stress is provided by a natural event, i.e. blooms of a toxic species, which likely affects the animals through the toxins produced. While direct impacts of *Ostreopsis* blooms on animal health^30^,^31^,^32^,^33^ and, more generally, of harmful algal blooms on invertebrates and vertebrates^34^, are well known, this study provides the first case of an indirect and delayed effect of a toxic bloom on the marine fauna. Despite the presence of relatively high toxin levels, animals exposed to *O.* cf. *ovata* blooms in July were apparently healthy. The stress status was, however, evident in the immature gonads from the high levels of NO, which has been shown to be a powerful stress indicator in *P. lividus*^24^,^25^,^35^ as well as in other marine organisms^36^,^37^,^38^ exposed to several stress agents. At the beginning of the reproductive period, i.e. in October, toxins were no longer detected but NO levels in the gonads were still high, while markers of the reproductive status, including spawning and fertilization success, were altered. In the period immediately preceding the bloom (June), as well as several months after the bloom (February), NO levels in the gonads were comparable to those measured in animals from the control area, supporting the idea that the observed alterations were due to the bloom of the toxic dinoflagellate.

Reproductive impairment was most and longer evident in the offspring of sea urchins collected in the reproductive period in the bloom area. Morphological anomalies were marked and frequent in the progeny obtained in October, a few months from the bloom, but were still conspicuous in April animals, affecting up to 37% of the plutei, supporting the idea of a transmission of the effects of the toxic alga from the mother to the progeny that develops in the following reproductive season. Maternal effects were also observed in the progeny of female sea urchins exposed to cadmium and manganese, which showed higher percentages of abnormalities than those obtained when only embryos were exposed to metals^25^. However, in that study the exposure to metal ions concerned sexually mature animals.

Morphological alterations and developmental failures had already been observed exposing sea urchin gametes and zygotes^14^ or larvae and juveniles^13^ to cultures of *Ostreopsis*, but these interactions presumably do not affect natural populations in the western Mediterranean Sea due to the mismatch between the *Ostreopsis* bloom and the reproductive period of *P. lividus*. By contrast, the delayed effects of the contact of the adults with toxic algae during the blooms presumably results in an impairment of the reproduction for a long part of the reproductive season at the MPA. A subsequent partial recovery observed in the population from April samples indicates that the impairment is reversible to some extent.

Interestingly, the abnormal development of the sea urchin progeny was found to be associated to alterations in the expression profiles of several marker genes of different processes, such as stress response, skeletogenesis and multidrug efflux transport genes, which are probably activated to counteract the effect of the toxic agent. The major modifications of gene expression profiles were found in the offspring of females collected in October, while a slight reduction in the variations was observed in the following months. The presence of an orchestrated gene defensome has already been observed in sea urchins as a response to external perturbation by various agents, including other algal toxins^39^,^40^, metal ions^24^,^25^, temperature^41^ and ocean acidification^42^. In April, alterations were still present in the expression profile. However, the pattern was different from that recorded in the progeny of October and February animals, suggesting that the alterations observed were rather associated with the recovery process, as supported by the lower percentage of abnormal embryos.

A noteworthy outcome of this study is the finding that NO acts as a potential agent underlying the reproductive impairment of the adults and the developmental abnormalities of the progeny of *P. lividus* in the study area. We believe that a critical step in the NO involvement is the nitration of the toposome, which is a major constituent of the yolk granule^43^. During embryogenesis, toposome follows a programmed proteolytic pathway that results in the generation of specific polypeptides^44^,^45^, which are supposed to transmit positional information during the early developmental stages^46^. Toposome nitration could lead to variations in the proteolytic pathway as also reported for other proteins and/or cause functional modifications of the protein^47^. The possible effect of the observed nitration on toposome functionality and its implications for embryo development in the progeny deserves deeper investigation.

**Figure 6:**
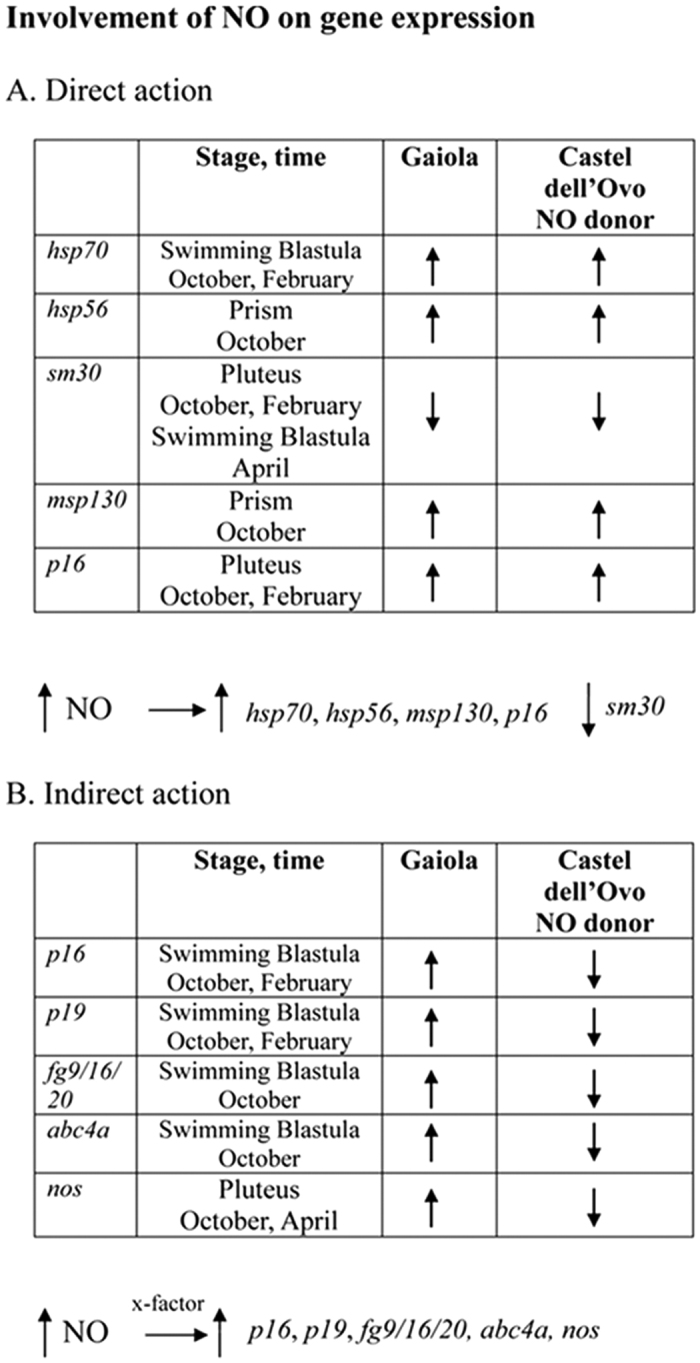
Schematic summary of the NO involvement in the modulation of gene expression in the offspring development of *P. lividus* from the MPA Gaiola. The expression of selected genes was examined in natural conditions at Gaiola site, where sea urchin progeny was characterized by high endogenous NO levels, in comparison to the progeny of animals from the control site to which NO was added experimentally. (**A**) Genes showing consistent regulation patterns in the two conditions, suggesting a direct regulation of NO (**B**) Genes showing opposite regulation patterns, suggesting an indirect regulation of NO.

There were also remarkably high levels of NO in the offspring of sea urchins from the study site impacted by *Ostreopsis* blooms, thus suggesting the NO role in the observed altered gene expression profile. Indeed, the addition or subtraction of NO under experimental conditions showed significant effects on the expression of the same genes that were altered under natural conditions at the study site. For a number of those genes (*hsp70, hsp56, sm30, msp130*), a regulation pattern similar to that observed at the study site was detected in the progeny of females from the control area upon exposure to a NO donor, indicating a direct control of NO on these genes (Fig. 6). For other genes (*p19, fg9/16/20, abc4a* and *nos*) the trend was opposite, suggesting an indirect control of NO on those genes through the intervention of unknown NO-regulated factors induced by the presence of the stress agent. An opposite pattern was also observed for the gene *p16* over the development in October and February, which indicated a direct NO-regulation at the pluteus stage and an indirect NO-regulation at the swimming blastula stage.

Overall these data indicate a very complex pattern of NO action on gene expression under a stress imposed by a biological agent, the toxic microalga, in the natural environment. Interestingly, *hsp70, sm30, p16, p19* and *fg9/16/20* were also identified as NO-target genes in sea urchin embryos exposed to decadienal or metal ions under experimental conditions^24^,^35^, and they showed a comparable complexity in their NO-regulated response to those stress agents, with direct and indirect regulation patterns. Whether the NO increase is a positive or negative agent in the animal response is a matter of debate. While the worsening of the development defects under reduced NO levels suggests a protective role of NO through the activation of several defense genes, NO can also be deleterious for the possible consequences of toposome nitration on development. Indeed, the dual role of NO has been reported in different processes, e.g. metamorphosis, cerebral ischemia and apoptosis^22^,^48^. Detrimental effects would be produced at high NO levels, which generate more reactive compounds, e.g. the nitrating agent peroxynitrite, whereas the effects would be positive at lower NO level increase.

**Figure 7:**
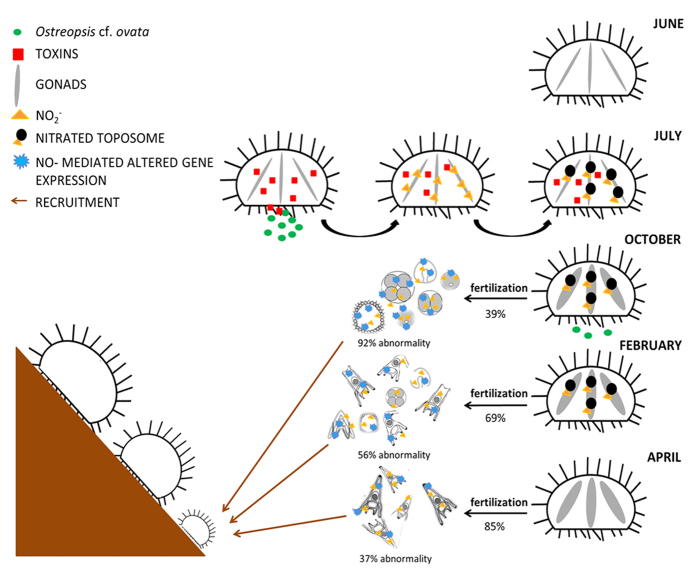
Proposed model of the processes involving *P. lividus* at the Gaiola MPA. Sea urchins subjected to intense summer blooms of *Ostreopsis* cf. *ovata* show alterations in their gonads and in several aspects of the reproductive process, as well as in the development of their progeny following the bloom. In July, the toxic agent, presumably acting through the ingestion of the epiphytic microalgae living on macroalgae as epiphytes, causes toxin accumulation and toposome nitration in the gonads of the immature sea urchin population. In the following seasons, toposome nitration persists in the mature gonads, while fertilization and developmental processes show impairments that decrease over time, yet not attaining a complete recovery. The expression of several marker genes is also altered in the sea urchin progeny, as a consequence of the increased NO levels. The reproductive impairment likely affects the recruitment potential of this key-species, with foreseeable consequences on adult populations and on the whole benthic ecosystem.

In summary, the effects of *O.* cf. *ovata* blooms are subtle in the apparently healthy adult sea urchins but become conspicuous during the reproduction and in the course of offspring development, through a series of events that are schematised in Fig. 7. At the time of the toxic bloom, sea urchins ingest epiphytic microalgae while feeding on macroalgae and accumulate toxins in their soft tissues. NO, determined as endogenous nitrite (NO_2_^−^) levels, increases in the gonads of exposed animals and is likely responsible for toposome nitration, as indicated by the comparison with animals collected prior to the bloom, which show low NO levels and no toposome nitration. In the reproductive season, several months after the toxic bloom, endogenous NO_2_^−^ levels are still high and the toposome is nitrated. The animals show a low fertilization rate and produce an abnormal offspring, with a likely reduced larval fitness in terms of growth and survival. The expression profile of key genes of stress, skeletogenesis and multidrug efflux is considerably altered in the offspring, presumably due to a defense response mediated by NO. Later in the season (April), NO_2_^−^ in gonads reverts to physiological levels, toposome is not nitrated and *P. lividus* produces a “better quality” progeny. However, alterations in the expression profile are still evident probably due to the recovery process.

## Conclusions

This study highlights a possible mechanism of transmission of the effects of a stressor across generations, by which the direct modification of the maternal gonads through NO action is at the origin of developmental impairments in the progeny occurring months after the stressful event. This mechanism could take place also in other benthic animals under multiple environmental pressures. The present study case suggests that HABs of *O.* cf. *ovata*, besides the known direct and conspicuous damages exerted in some cases, may have hidden and sublethal outcomes for *P. lividus*, with “carry-over” consequences for reproduction and offspring development and related cascade effects, which can constitute relevant bottlenecks for natural populations. The partial recovery at the time of the reproductive peak of sea urchin may limit the negative consequences of the toxic blooms depicted above. Yet, considering all sorts of other pressures to which this fundamental species is exposed, the HAB-related impairment of reproductive ability, its impact on recruitment and the possible reverberation across the benthic system should be taken into account in the evaluations of the health of marine coastal environments.

## Methods

### Study area

The study area is located in the urban area of Naples (Tyrrhenian Sea, Mediterranean Sea), along the coast of Campania Region (Supplementary Fig. S1). The two sampling sites, Gaiola MPA and Castel dell’Ovo are at a distance of 3.45 nm (nautical miles) on a direct beeline. Gaiola is included in an MPA (41.6 ha) established in 2005 in an area of great geological, environmental and archaeological importance. The MPA includes two zones: an “A zone” of strict nature reserve, where *Paracentrotus lividus* and *Ostreopsis* cf. *ovata* were sampled, and a “B zone” where fishing and recreational activities are allowed but regulated (more details here http://www.areamarinaprotettagaiola.it/Mappa_AMPGaiola.pdf). The MPA Gaiola has been interested by very low anthropogenic activity over centuries, maintaining to some extent its original environmental features^49^. The control site Castel dell’Ovo is closer to the Naples city centre and hosts one of the few other *P. lividus* populations of the area. Mean values of the main environmental variables (temperature, salinity, nutrients) in summer are similar between the two sampling sites, although slightly higher nutrient values and wider salinity fluctuations are recorded at Castel dell’Ovo. For example, in the latter site dissolved inorganic nitrogen was 2.24 ± 1.82 μM (42 data points 2001–2005, www.sidimar.it) compared to 1.71 ± 1.06 μM recorded for MPA Gaiola (92 data points, summer 2007–2014)^50^.

At the MPA Gaiola site, *O.* cf. *ovata* is always present and regularly blooms intensively on summer (over an 8 year study since 2007)^50^. By contrast, at the Castel dell’Ovo site, *O.* cf. *ovata* was generally found at very low concentrations (<20 cells g^−1^ macroalgal fw, from July to September) in the regional monitoring program started in 2007 (http://burc.regione.campania.it). Therefore the monitoring at that site was interrupted from 2009^51^,^52^. Low levels of *O.* cf*. ovata* densities (<80 cells g^−1^ macroalgal fw) were also recorded at the Castel dell’Ovo sampling site during October 2013.

### Ethics statement

*Paracentrotus lividus* (Lamarck) sea urchins were collected in the Gulf of Naples at the Gaiola MPA at station G1 (40°47.494’N, 14° 11.282’E), with the authorization of the Soprintendenza Speciale per i Beni Archeologici di Napoli e Pompei. Control animals were collected in the Gulf of Naples, near Castel dell’Ovo (Lat. 40°49’ 41”, Lon. 14°14'48”) from a location that is not privately-owned or protected in any way, according to the authorization of Marina Mercantile (DPR 1639/68, 09/19/1980 confirmed by D. Lgs. 9/01/2012 n. 4). The field studies did not involve endangered or protected species. All animal procedures were in compliance with the guidelines of the European Union (directive 2010/63 and following D. Lgs. 4/03/2014 n. 26).

### *Ostreopsis* sampling

Sampling of benthic microalgae was carried out at Gaiola MPA previous to the bloom (June 2012), during blooms (July 2012 and 2014) and in the following months (October 2013, February and April 2014), in the framework of the “*Ostreopsis* Monitoring Plan” for the Campania Region (Italy). Microalgal epiphytes were obtained from triplicate samples of the red algae *Asparagopsis taxiformis, Halopteris scoparia* or *Jania rubens* collected by SCUBA diving at a depth of ca. 4.5 m using plastic bags. Macroalgal samples were processed for the detachment of epiphytic *Ostreopsis*^12^. *Ostreopsis* cells were counted following the Utermöhl method^53^. Cell abundance values were expressed as cells g^−1^macroalgal fw.

### Sea urchin collection, maintenance and handling

*P. lividus* (50 animals/sampling), with mean size of 4.5 ± 1.5 cm in diameter, were collected by SCUBA divers at Gaiola MPA in different periods of the year concomitant to *O.* cf. *ovata* sampling. Parallel to the sampling at Gaiola, control sea urchins were collected at Castel dell’Ovo, where *O.* cf. *ovata* is present at very low concentration. Animals were transported in insulated boxes to the laboratory within 1 h and maintained in tanks with water from Gaiola or from control sites, at least for 1 night. The animals were visually examined to identify possible negative effects on their health.

### Toxicity tests

The toxicity tests, including toxin extraction and determination by LC-MS TOF^4^ were performed at the Istituto Zooprofilattico Sperimentale del Mezzogiorno, Portici, Napoli, following the D.M. del 16/05/2002 – Protocol 2-Step 2, within the monitoring project Ostreopsis Regione Campania. In addition, samples collected at Gaiola and Castel dell’Ovo in July 2012, 2013 and October 2013, were also used for toxin determination.

### Gonads collection, determination of GSI, spawning and fertilization success

Females were identified by microscopic observation. Ovaries were removed, weighed, washed with PBS, frozen in liquid nitrogen and kept at −80 °C until analysis. Eggs were collected and fertilized as previously described^24^. The GSI value of the females was calculated as the ratio of the gonad mass to the whole-body wet mass (%). The spawning was determined as the ratio between the number of spawning females and the total number of females (%). The fertilization success was calculated as the ratio of the fertilized eggs observed at the first division (1 h) respect to the number of total eggs.

### Gamete collection, embryo culture and morphological analysis

To collect eggs, gonads were gently washed in sea water to allow their release and fertilized as previously described^24^. The fertilization success in control samples was approximately 90%. Fertilized eggs (150 eggs/ml) were allowed to develop in a controlled temperature chamber at 18 ± 2 °C and 12:12 light:dark cycle. The development was followed by inverted microscope (Zeiss Axiovert 135 TV) until the pluteus stage, approximately 48 hours post fertilization. Embryos were fixed in 4% formalin and morphological observations were performed to identify eventually abnormal plutei. Embryos were considered normal if they reached the pluteus stage of development, exhibited good body symmetry, showed fully developed skeletal rods and displayed a well differentiated gut. All the morphologies that did not satisfy the above-mentioned criteria were grouped and referred to as abnormal^24^.

### NO determination

Endogenous NO levels were measured by monitoring nitrite formation with the Griess reaction^24^. Collected ovaries, washed in PBS and frozen in liquid nitrogen, were homogenized in 20 volumes of PBS and centrifuged at 25,000 × g for 20 min at 4 °C. The supernatants were analyzed for nitrite content. Sea urchin developing embryos were collected at different developmental stages and analyzed for nitrite content^24^.

### Protein extraction and immunoblotting

Briefly, ovaries were homogenized on ice in 20 volumes of 10 mM Tris-HCl buffer containing 10 mM NaCl at pH 8.0, supplemented with 0.1 mM phenylmethylsulfonylfluoride (PMSF)^54^. Lysates were centrifuged at 25,000 × g for 20 min at 4 °C. Supernatants were collected and total protein concentration was determined using a Bio-Rad Protein Assay Reagent (Bio-Rad, Milan, Italy) with bovine serum albumin as a standard. Before electrophoresis, protein samples used for Coomassie staining were incubated at 85 °C for 5 min, whereas samples examined for nitrated proteins were not. Equal amounts (10 μg) of protein extracts were separated by SDS-PAGE under reducing conditions on 7.5% gels. Following SDS-PAGE, gels were stained with Coomassie or blotted onto polyvinylidendifluoride (PVDF, Sigma-Aldrich) membrane. To detect nitrated proteins, the membrane was analyzed with monoclonal anti nitrotyrosine antibody (1:20,000) (Invitrogen). After washing in 20 mM Tris pH 7.6, 137 mM NaCl and 0.1% Tween 20, labeled proteins were detected by Amersham ECL Prime Western Blotting Detection Reagent (GE Healthcare, EuroClone, Milan, Italy).

### Liquid Chromatography Electrospray Tandem Mass Spectrometry (LC–ESI-MS/MS)

For protein identification, sea urchins ovaries were collected and the protein extracts were digested by trypsin sequence grade upon precipitation in 10% TCA (trichloroacetic acid), reduction with 45 mM dithiothreitol and alkylation with 100 mM iodoacetamide. MS/MS analysis was carried out by a LTQ-Orbitrap Velos (Thermo Fisher Scientific), as previously described^55^. Data Base searching was performed using the Sequest search engine of Proteome Discoverer 1.1 (Thermo Fisher Scientific).

### Gene expression analysis

Fertilized eggs (about 1500) were collected by centrifugation. Total RNA was extracted from each developmental stage using RNAqueous-Microkit (Ambion) according to the manufacturer’s instructions and quantified by UV absorbance as described previously^24^. For each sample, 600 ng of total RNA extracted was retrotranscribed with iScriptTM cDNA Synthesis kit (Biorad), following the manufacturer’s instructions. cDNA diluted 1:5 with H_2_O was used in Real Time qPCR experiments as described previously^24^. The data were normalized for the reference gene *PlZ12-1*, which resulted to be the most suitable reference gene in previous studies^24^,^25^,^56^,^57^,^58^,^59^,^60^.

### Pharmacological treatments

Pharmacological treatments with the slow releasing NO donor (Z)-1-{N-(3-Aminopropyl)-N-(4-(3-aminopropylammonio)butyl)-amino}-diazen-1-ium-1,2-diolate) (spermine NONOate, sperNO) (Alexis, San Diego, California), spermine, the product derived from sperNO after NO release (Sigma–Aldrich, Milan, Italy) and NOS inhibitor 1-(2-trifluoromethylphenyl)imidazole (TRIM) (Alexis, San Diego, California) were performed as described previously^24^.

### Statistical analysis

Data were analyzed for normality and homogeneity of variance with the Shapiro-Wilk test and Levene’s test, respectively. For the reproductive state (GSI and fertilization success) and offspring morphology, a two-way ANOVA with Bonferroni post hoc test was performed to determine how the biological responses were affected by sampling time and site. Differences in immunopositive bands obtained from densitometric analysis were tested through a one-way ANOVA with Dunnett’s post hoc test. A non-parametric Kruskal-Wallis test (K/S) with Mann-Whitney pairwise as post hoc test was used for the variable “position” that did not satisfy normality assumption for nitrite levels and spawning data. For all tests, significance level was set for p < 0.05. Statistical analysis were performed with Past software ver. 3.11 (http://folk.uio.no/ohammer/past/terms.html). Graphics were created with GraphPad Prism 4.0 for Windows (Graphpad Software, San Diego, CA, USA). For Real Time qPCR analysis, significance was tested using the “Pair Wise Fixed Reallocation Randomisation Test”, developed by REST^61^.

## Additional Information

**How to cite this article**: Migliaccio, O. *et al*. Subtle reproductive impairment through nitric oxide-mediated mechanisms in sea urchins from an area affected by harmful algal blooms. *Sci. Rep.* **6**, 26086; doi: 10.1038/srep26086 (2016).

## Supplementary Material

Supplementary Information
