# Supplementary material for: Subtle reproductive impairment through nitric oxide-mediated mechanisms in sea urchins from an area affected by harmful algal blooms
Source: Sci Rep. 2016 May 19;6:26086. doi: 10.1038/srep26086 (PMC4872146; doi:10.1038/srep26086)
Supplement: Supplementary Information [file srep26086-s1.pdf]

Subtle reproductive impairment through nitric oxide-mediated mechanisms in sea urchins from an area affected by harmful algal blooms

Oriana Migliaccio, Immacolata Castellano, Davide Di Cioccio, Gabriella Tedeschi, Armando Negri, Paola Cirino, Giovanna Romano, Adriana Zingone & Anna Palumbo

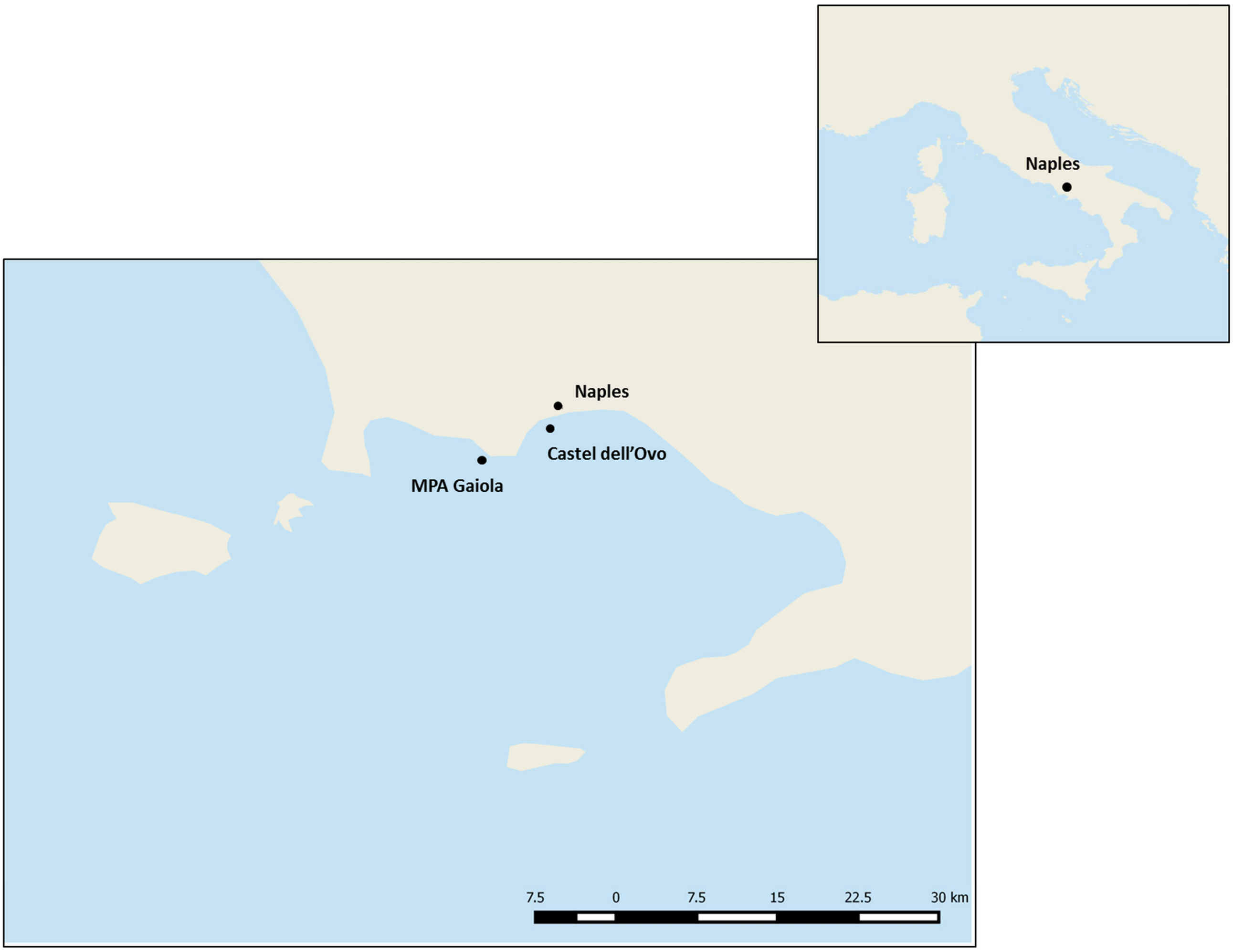

Supplementary Fig. S1. Map of the study area generated with QGIS 2.12 (http://qgis.org/downloads/)
